# Novel nerve regeneration assessment method using adult zebrafish with crush spinal cord injury

**DOI:** 10.1007/s00359-024-01723-4

**Published:** 2024-11-12

**Authors:** Hiroaki Motohashi, Satoshi Sugita, Yoshito Hosokawa, Takahiro Hasumura, Shinichi Meguro, Noriyasu Ota, Yoshihiko Minegishi

**Affiliations:** Biological Science Research, Kao Corporation, 2606 Akabane, Haga-gun, Ichikai-machi, Tochigi 321-3497 Japan

**Keywords:** Fig, Nerve regeneration, Spinal cord, Zebrafish

## Abstract

Zebrafish (*Danio rerio*), an alternative to rodents, are widely used in neurological, genetic, and toxicology research. The zebrafish larval spinal cord injury model has been used in neural mechanistic analyses owing to its high regenerative capacity and throughput; however, it also had several limitations in imitating rodents. Therefore, we investigated the use of adult zebrafish as an alternative model to rodents for evaluating nerve regeneration. Here, we established a novel spinal cord regeneration evaluation method, which was based on the maximum swimming speed of adult zebrafish in a custom-built hydrodynamic-based aquarium. The spinal cords of adult male zebrafish were crushed using forceps, and maximum swimming speed and histological spinal cord regeneration were evaluated. Spinal cord-injured zebrafish showed a significant decline in motor function, followed by recovery at 3 weeks postoperatively, accompanied by histological regeneration. Spinal cord regeneration can be indirectly assessed by monitoring maximum swimming speed. They were also fed a diet containing fig extract, which can promote peripheral nerve regeneration; they were fed daily starting 1 week before the operation. Maximum swimming speed was measured time-dependently until 3 weeks postoperatively. Fig-consuming fish showed improved recovery of maximum swimming speed compared to the controls, which was consistent with the histological analysis. In summary, we established a spinal cord regeneration assessment system using adult zebrafish in a customized aquarium, which enables researchers to evaluate spinal cord regeneration in adult zebrafish similar to that of rodent experiments, contributing to faster and easier screening of neuroregenerative technology.

## Introduction

Animal testing using rodents plays an important role in the research and development of drugs and healthcare products that improve human lives. Recently, alternative methods for rodent experiments have been investigated globally with the purpose of improving the efficiency of research and animal welfare. Based on the 3R philosophy (Replacement, Reduction, Refinement) (Russell and Burch 1959), the usefulness of zebrafish (*Danio rerio*) as a model organism in enhancing the efficiency of animal research has gained increasing attraction.

Zebrafish are classified as vertebrates and are widely used in neurological, genetic, and toxicological studies (Patton et al. 2021; Stewart et al. 2014). They share more than 70% genomic information with humans (Howe et al. 2013; Postlethwait et al. 1998), and can be easily genetically manipulated by developing genetic target systems represented by CRISPER/Cas9 (Choi et al. 2021). Therefore, they have proven to be an effective model in biological studies that apply genetic techniques. One of the biological characteristics of zebrafish is their high regenerative capacity. The central nervous system (CNS) of zebrafish, the brain and spinal cord, regenerates after injury (Becker et al. 1997; Kroehne et al. 2011). To date, some mechanisms of regeneration in the injured part of the zebrafish CNS have been clarified. These include the differentiation of ependymal brain stem cells into new neurons and the formation of glial bridges, which are promoted by growth factors secreted in the injured area (Cigliola et al. 2023; Klatt Shaw et al. 2021; Mokalled et al. 2016; Shimizu et al. 2018).

Zebrafish studies typically use larvae because of their high throughput, and their evaluation results are consistent with those of rodents and humans (Rennekamp and Peterson 2015)_._ For example, in a neurological study, tranexamic acid, which was shown to be effective for spinal cord regeneration in a mouse model of crush spinal cord injury (cSCI), was screened using a larval spinal cord transection model (Chapela et al. 2019). Larvae exhibit advantages such as increased throughput and are considered as non-animal until 5 days post fertilization (dpf) (EFSA 2005). However, in vivo level data, including histological analysis and phenotypes of motor functions such as swimming speed, are lacking. Moreover, the zebrafish immune system, which is pivotal for neural regeneration, matures functionally and morphologically by 4–6 weeks post fertilization (wpf) (Lam et al. 2004); thus, zebrafish larvae have an immature immune system. Therefore, compared to rodents, zebrafish larvae present limitations regarding functional assessment and immunity.

Adult zebrafish have a higher throughput than that of rodents; however, it is not as high as that of larvae. Moreover, in vivo level data can be obtained with adult zebrafish, which was not possible with larvae. Zebrafish, adult and larvae, are capable of CNS regeneration, and many mechanisms have been reported using spinal cord transection and cSCI models (Klatt Shaw et al. 2021; Mokalled et al. 2016; Tsata et al. 2021; Wehner et al. 2017).

Regeneration after cSCI in adult zebrafish is complicated; however, macrophage/microglial reactions and cross-linking of glial cells after spinal cord transection have been reported, which are very similar to those performed by Schwann cells in the peripheral nerves of rodents and humans (Abe et al. 2020; Bunge 1994; Min et al. 2021).

*Ficus carica* L. (fig) is a fruit rich in bioactive compounds, including polyphenols, carotene, and vitamins. Fig by-products provide health benefits against many diseases such as cancer, diabetes, and cardiovascular disease (Rasool et al. 2023). Moreover, owing to its antioxidant and neuronal regeneration effects, dietary polyphenol intake has been gaining attention as a therapeutic option for neurodegenerative diseases such as Parkinson’s disease and Alzheimer’s (Ataie et al. 2016).

In our previous study, fig extracts contribute to peripheral nerve regeneration (Sugita et al. 2024). Continuous administration of fig extract mixed with food to sciatic nerve-injured mice improved nerve conduction velocity and restored the myelin sheath. In addition, the number of macrophages that aggregate in the injured part of the nerve increased after fig administration, suggesting that it promotes peripheral nerve regeneration through the facilitation of immune responses (Sugita et al. 2024). Detailed analyses of zebrafish macrophage subtypes revealed that zebrafish immune systems are very similar to those of mammals (Meeker and Trede 2008) and maintain a similar population (Nguyen-Chi et al. 2015), with some differences (Geirsdottir et al. 2019). Because the zebrafish spinal cord and mouse peripheral nerves seem to share key mechanisms, we considered the possibility that this material could be effective in both models.

In this study, we aimed to establish a spinal cord regeneration assessment system using adult zebrafish and investigate the efficacy of fig extract in promoting spinal cord regeneration in the model. We hypothesized that the adult zebrafish model could serve as an effective alternative to rodent models for studying peripheral neuropathy, given their shared partial regeneration mechanism. We adopted the cSCI model for this study because it allows for swim function recovery within 4 weeks (Hui et al. 2010), whereas the transection model requires more than 6 weeks (Cigliola et al. 2023; Tsata et al. 2021).

## Materials and methods

### Animals

Adult male Riken Wildtype (RW) zebrafish (*Danio rerio*) were the offspring of zebrafish transferred from the RIKEN Center for Brain Science (Japan). Transgenic zebrafish *Isl1-*GFP (Higashijima et al. 2000), which express green fluorescent protein (GFP) in motor neuron cells/sensory ganglion cells, were also obtained from the RIKEN Center for Brain Science (Japan) with support from the National Bio Resource Project (NBRP). *Mbp*:EGFP-CAAX (Almeida et al. 2011), expressing the EGFP-CAAX myelin marker in myelin basic protein (MBP), was used in this study. The *Mbp* promoter was obtained from larval genomic DNA of RW by PCR using forward primer 5′-GTTGATCTGTTCAGTGGTCTACAG-3′ and reverse primer 5′-CAGATGCTGAGATGTGACTACTGC-3′. After confirming that the target region was appropriately amplified by DNA sequencing, the promoter was replaced with the elongation factor 1-alpha (EF1α) promoter intron of pT2AL200R150G, using PCR amplification and an In-Fusion (Takara) reaction for transgenesis with the Tol2 transposon system. To insert DNA sequence encoding CAAX motif between the EGFP sequence and stop the codon to visualize the myelin sheath, the Tol2 plasmid with the *Mbp* promoter was amplified using the forward primer 5′-AAGCTGAACCCTCCTGACGAGAGCGGACCTGGATGCATGAGCTGCAAGTGCGTGCTGAGCTAAAGCGGCCGCCACCGCGG-3′ and reverse primer 5′-TCGTCAGGAGGGTTCAGCTTCTTGTACAGCTCGTCCATGC-3,’ and the In-Fusion reaction was initiated. The Tol2 plasmid vector and Tol2 transposase mRNA were microinjected into 1-cell stage embryos. At 3–5 dpf, the injected larvae that were positive for EGFP signals in the CNS were screened and raised to establish a stable transgenic line. F3 or later generations of transgenic fish were used for the experiments. All fish were housed at 28 ± 1 ℃ with 55% ± 10% humidity under a 14 h light and 10 h dark cycle. Water quality conditions were maintained according to The Zebrafish Book (Westerfield 2007). All experiments were conducted in accordance with the Animal Care and Experimentation Facility Committee of Kao Corporation (Tochigi, Japan) and outlined in the Guide for the Care and Use of Laboratory Animals, 8th edition (Council 2011).

### Crush SCI

cSCI was induced at the dorsal fin level with 0.0075% (w/v) tricaine (Sigma-Aldrich, St. Louis, MO, USA), based on a previous study (Hui and Ghosh 2016; Hui et al. 2010, 2014). After cutting 3 mm around the injury site with a razor blade, the spinal cord was crushed with #5 Dumont forceps for 1 s. In the Sham group, only cutting was performed. All surgical procedures were conducted under a microscope, and the fish were kept in a 4.27 μM of methylene blue (JAPAN PET DESIGN CO., LTD., Japan) for at least 2 days to prevent infection from the operative wounds.

### Fig extracts

As described in our previous study (Sugita et al. 2024), fig extracts were prepared from 500 g dried fig, which were soaked in 5 L of 99.5% ethanol for 8 d at room temperature. The mixture was then filtrated, concentrated, diluted with water, and lyophilized. The resulting fig-containing diet comprised 3% fig incorporated into Otohime B2 (Marubeni Nisshin Feed Co., Tokyo, Japan). The fig extract, dissolved in water, was mixed with Otohime B2, freeze-dried for more than 12 h, and subsequently combined with lard to mask any flavor preferences. The ratio of lard to diet was maintained at 20%, consistent with a normal diet.

The fig-containing diet was administered twice daily, with an approximate food intake of 8 mg/day per individual. Each portion of the diet was scattered in the tank to allow for free feeding, similar to the normal feeding regimen.

### Swim capacity assays

**Fig. 1:**
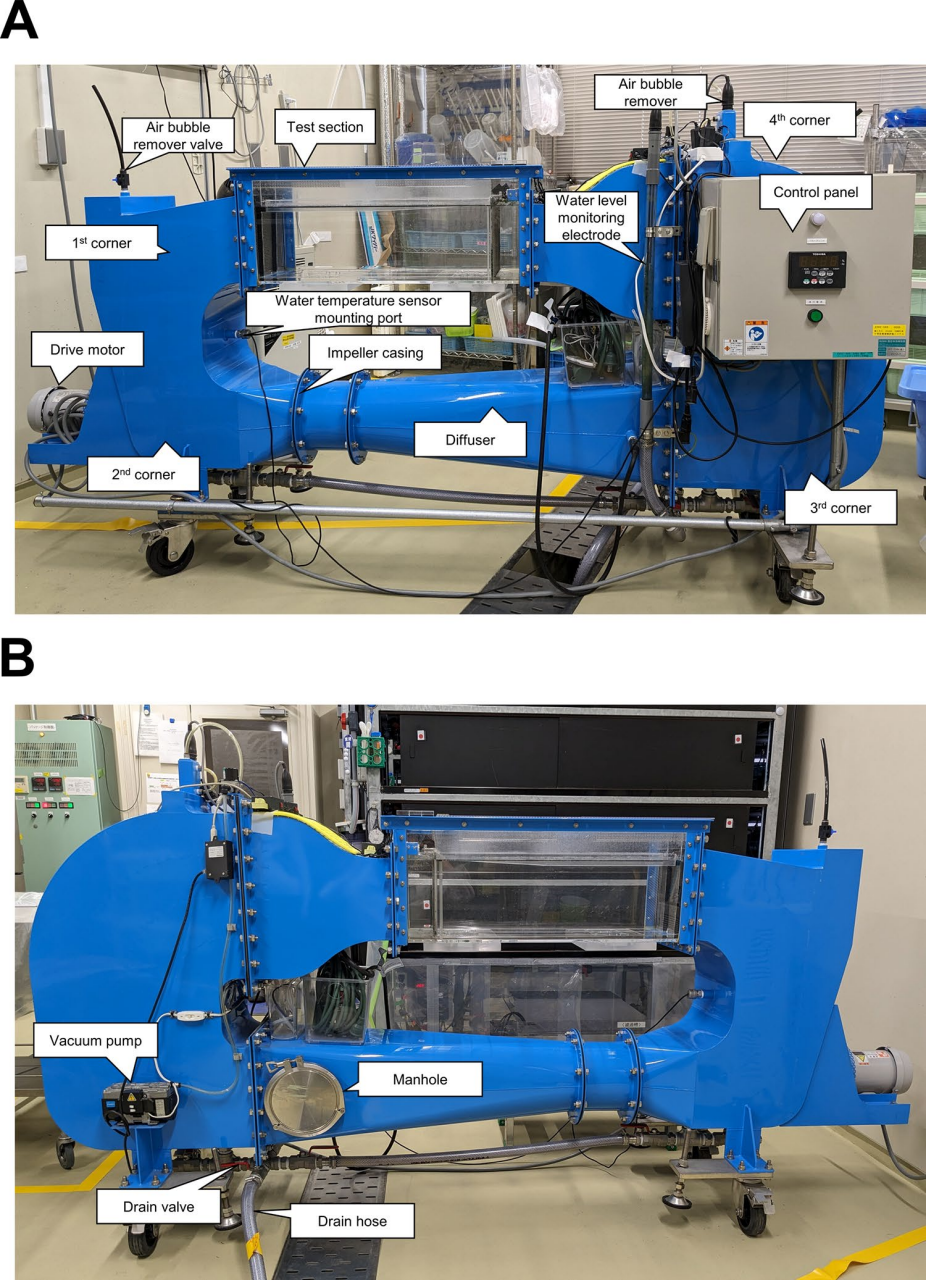
Overall view of the modified PT-70S and names of each part. **a** Control panel-side of the modified PT-70S. **b** Manhole-side of the modified PT-70S

**Fig. 2:**
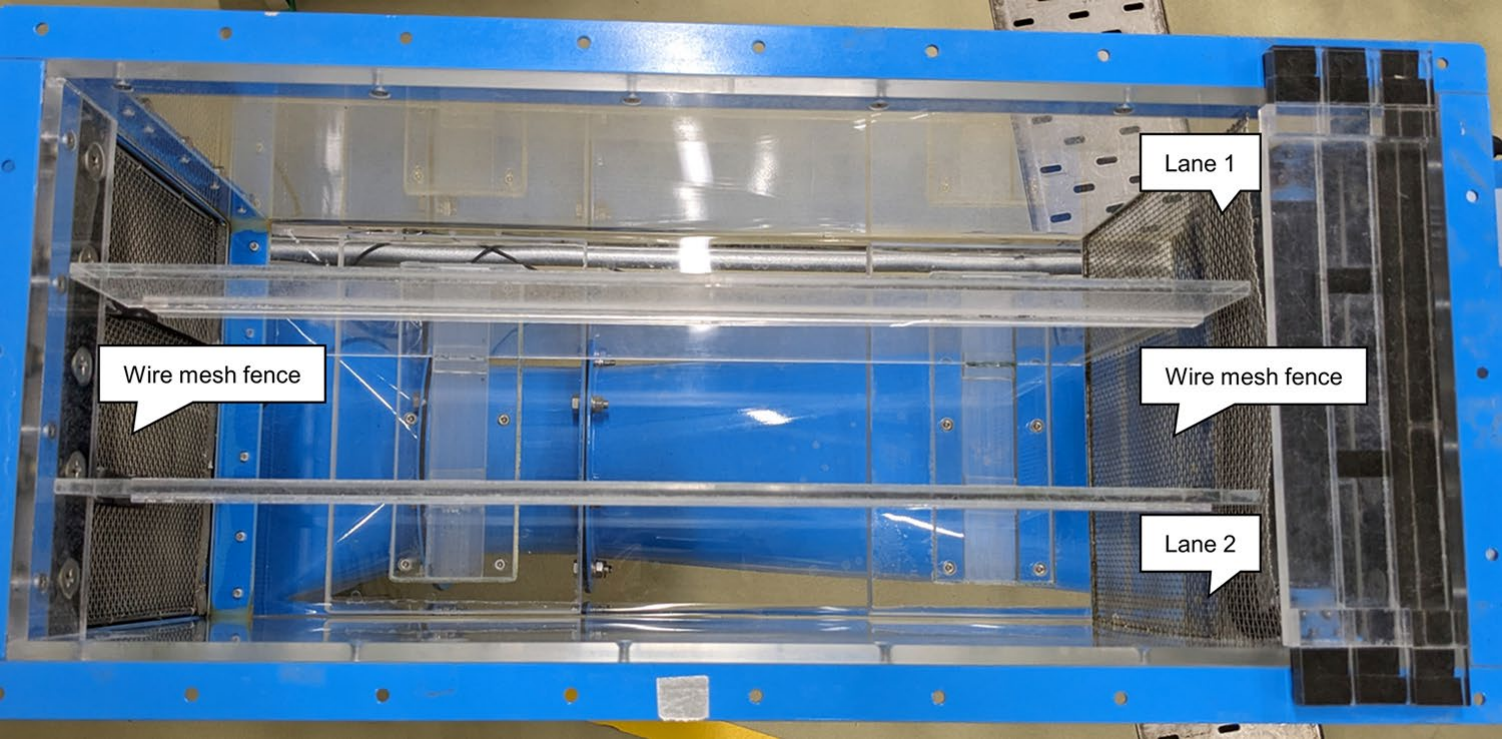
Overhead view of the test section of the modified PT-70S (from the control panel-side)

Maximum swim speed was continuously assessed in a spinal cord regeneration test. We used the swim capacity assay 320 L Brett-style recirculating swim flume modified from the Personal-Tank PT-70S (West Japan Fluid Engineering Laboratory Co., Sasebo, Japan), as reported in our previous study (Hasumura and Meguro 2016). This tank had three swim courses (70 cm long, 10 cm wide, and 30 cm high) and could evaluate 10 fish simultaneously in each course. An overall view of the tank, along with the names of each section, are depicted in Figs. 1A, B, and 2. Detailed specifications of the aquarium are presented in Table 1. Prior to the assays, the fish were transported to lanes 1 and 2. After acclimatization for 10 min at 1 Hz, the speed was increased by 1 Hz every 1 min (1.4 Hz = 4.5 cm/s). Maximum speed was defined as the speed at which the fish could not swim. An observer positioned at the side of the tank adjusted the water flow speed using a control panel and recorded the number of fish caught in the wire mesh at the rear. Exclusion criteria for operation failure was > 12 Hz in the first assay and > 20 Hz for the second assay post cSCI. One fish was excluded from the experiment (Experiment 4, cSCI group).

**Table 1:** Specification of the tank for swim capacity assays

	Model number	Modified PT-70S
Main body	Power supply	AC100V
	Power consumption [W]	825
	Water temperature [°C]	28±1
	Safety equipment	Power OFF with abnormal water levels
	L×B×H [mm]	2400×504×1158
	Sluicing device	Impeller and drive motor
	Flow velocity control	Inverter control
	Air bubble removal device	Vacuum pump type
Test section	L×B×H [mm]	700×300×300
	Material	transparent acrylic resin
	Capacity of water [L]	320
	Maximum flow rate [cm/sec]	120
	Flow rate distribution	<10%
	Number of lanes	3

### Spontaneous activity assays

Spontaneous activity was continuously evaluated using infrared (IR) sensors. Each zebrafish was placed in an aquarium with a sensor in the middle, and the number of movements through the sensor was measured, which was defined as spontaneous activity. Measurements were performed continuously for 24 h, and activity at any hour during the light period was recorded. The sensor was fabricated using an optical fiber sensor with the fiber unit FU-A100 and amplifier FS-N11CN (Keyence Corporation, Japan). A 6-channel connected and displayable counter was used.

### Spinal cord regeneration analysis

Approximately 1.5 cm of the torso with the spinal cord was dissected, transferred into 4% paraformaldehyde phosphate buffer (Wako Pure Chemical Industries), and preserved at 4 ℃ overnight. Subsequently, they were embedded into O.C.T. compound (Sakura Finetek Japan Co., Ltd., Japan) and stored at −80 ℃. Thereafter, 50 µm thick sections were sliced using a microtome (Leica Microsystems GmbH, Germany), transferred to the slide glasses PRO-01 (Matsunami Glass Ind., Ltd., Japan), and washed twice with phosphate-buffered saline. The samples were sealed with a Vectashield Mounting Medium Hard Set (Vector Laboratories, Inc., CA 94560, USA). Microscopic observations were performed using fluorescent microscopy BZX700 (Keyence Co., Osaka, Japan). Spinal cord regeneration was evaluated by calculating the spinal cord regeneration rate using the ratio of the thickness of the injury site to that of the intact site. Histological analyses were performed as described previously (Stil and Drapeau 2016).

### Experimental design

#### Experiment 1

The fish were assigned to two groups (n = 8, 16 for sham and cSCI groups) based on their swim capacity. Both groups were fed a control diet twice per day from 1 week before to 3 weeks after cSCI induction, and swim capacity was monitored continuously. Otohime B2 (Marubeni Nisshin Feed Co., Tokyo, Japan) was used as the control diet. The food was freeze-dried for more than 12 h, then mixed with lard for coating to eliminate flavor preferences.

#### Experiment 2

The fish were assigned to two groups (n = 8, sham and cSCI groups) based on their body weight and spontaneous activity. All fish were housed in individual cages. The diet and feeding process were as that in Experiment 1. Body weight and spontaneous activity were monitored continuously.

#### Experiment 3

Transgenic zebrafish (*Isl1-*GFP and *Mbp*:eGFP-CAAX) were divided into two groups, fed a control diet, and subjected to cSCI as in Experiment 1. Longitudinal sections of the spinal cord from both groups were used to evaluate spinal cord regeneration. Fish were euthanized with excess tricaine (Sigma-Aldrich, St. Louis, MO, USA) and sections from the cSCI group at days 8, 14, 22, and 35 post-cSCI were evaluated for *Isl1-*GFP, and at days 0 and 35 post-cSCI for *Mbp*:eGFP-CAAX.

#### Experiment 4

The fish were assigned to three groups (n = 16; sham, cSCI, and cSCI + fig groups) based on body weight and swimming capacity. The sham and cSCI groups were fed the control diet, while the cSCI + fig group was fed a fig-containing diet twice per day from 1 week before to 3 weeks after cSCI induction. The body weight and swimming capacity were continuously evaluated for each group. The fig-containing diet contained 3% fig in Otohime B2 (Marubeni Nisshin Feed Co., Tokyo, Japan). Fig extract dissolved in water was mixed with Otohime B2, freeze-dried for more than 12 h, then mixed with lard for coating to eliminate flavor preferences.

#### Experiment 5

Transgenic zebrafish *Isl1-*GFP were assigned to three groups (n = 7, 19, 19 for sham, cSCI, and cSCI + fig groups, respectively) based on their swimming capacity. The sham and cSCI groups were fed the control diet, while the cSCI + fig group was fed a fig-containing diet twice per day from 1 week before to 2 weeks after cSCI induction. The body swimming capacity was continuously evaluated for each group. Fish were euthanized with excess tricaine (Sigma-Aldrich, St. Louis, MO, USA) at week 2 and longitudinal sections of the spinal cord from each group were provided for spinal cord regeneration evaluation. Data are presented by integrating the results from two separate experiments.

### Statistical analysis

All values are expressed as means ± standard error. The difference was considered significant at *p* < 0.05. Data were analyzed using the GraphPad Prism 6 J software (GraphPad Software Inc., San Diego, CA, USA). In the two-group comparison, normal distribution and equal variance were tested using the Kolmogorov–Smirnov test and F-test, respectively. For parametric analysis, a *t*-test was conducted as a post-hoc analysis. Nonparametric analysis was performed using the Mann–Whitney U test.

## Results

### Maximum swimming speed, body weight, and spontaneous activity in fish with cSCI

**Fig. 3:**
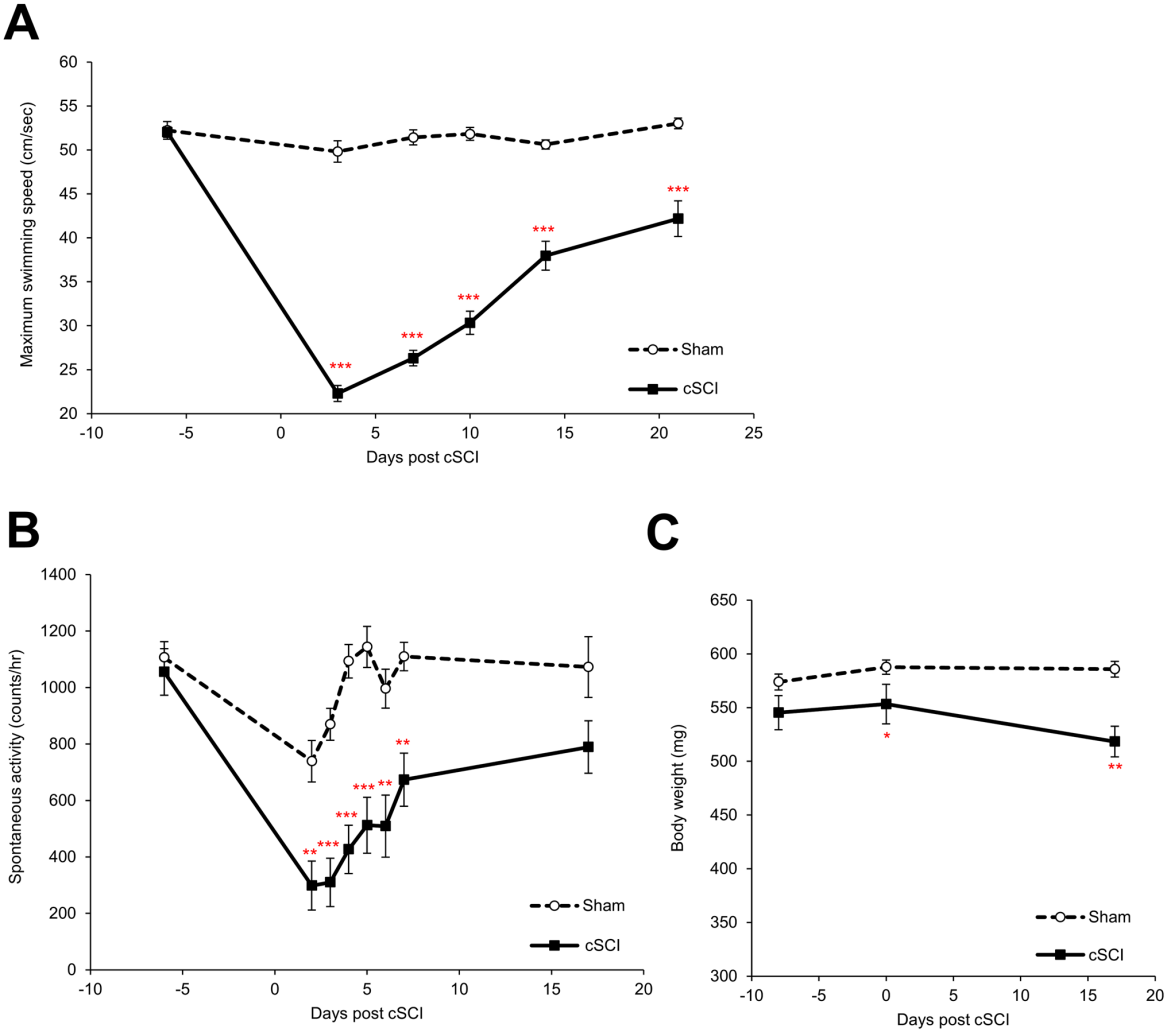
Maximum swimming speed, spontaneous activity, and body weight for the sham and cSCI zebrafish. **a** Time-course changes of maximum swimming speed for the sham and cSCI zebrafish. **b** Time-course changes of spontaneous activity for the sham and cSCI zebrafish.** c** Time-course changes of body weight for the sham and cSCI zebrafish. Values are expressed as means ± standard errors: *p < 0.05, **p < 0.01, ***p < 0.001 vs Sham

Maximum swimming speed of the cSCI zebrafish model was measured in Experiment 1. The maximum swimming velocity did not change significantly after surgery in the sham group but decreased significantly immediately after surgery in the cSCI group; however, they recovered to 80% of their preoperative levels by day 18 post cSCI (Fig. 3A). In Experiment 2, the spontaneous activity and body weight of each fish were monitored. The spontaneous activity dropped significantly after surgery in the cSCI group but recovered to 70% of the preoperative level on day 17 post cSCI (Fig. 3B). Body weight gain in the cSCI group was significantly lower than in the sham group postoperatively until day 17 post-cSCI (Fig. 3C).

**Fig. 4:**
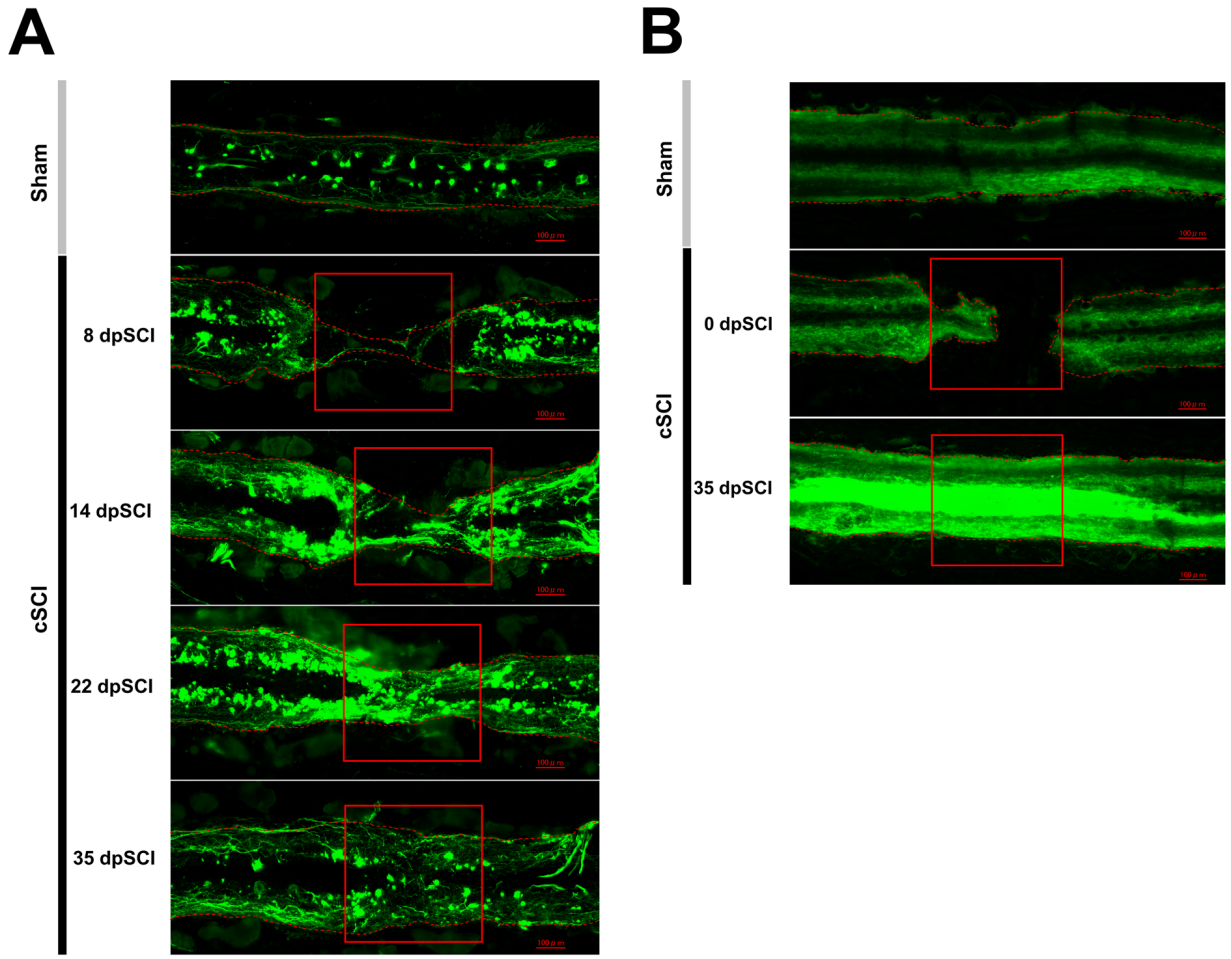
Time-course histological analysis of the injury site in the spinal cord. **a** Spinal cord histology for the sham and cSCI *Islet-1-*GFP zebrafish at 8, 14, 22, 35 days post-cSCI. **b** Spinal cord histology for the sham and cSCI *Mbp*:eGFP-CAAX zebrafish at 0 and 35 days post-cSCI. Red squares indicate the cSCI sites. Scale bars in **a** and **b** 100 μm

In Experiment 3, using transgenic zebrafish *Isl1-GFP* to visualize spinal motor neurons via GFP expression under the Isl1 promoter, we confirmed that the spinal cord was severely damaged by cSCI. Moreover, gradual regeneration occurred, and the connections at the damaged site became thicker (Fig. 4A). We also generated transgenic zebrafish, *Mbp*:EGFP-CAAX, to visualize the expression of myelin proteins which confirmed that the myelin sheath was cleaved but regenerated by day 35 post-cSCI (Fig. 4B).

### Fig extract administration boosts swim capacity recovery

**Fig. 5:**
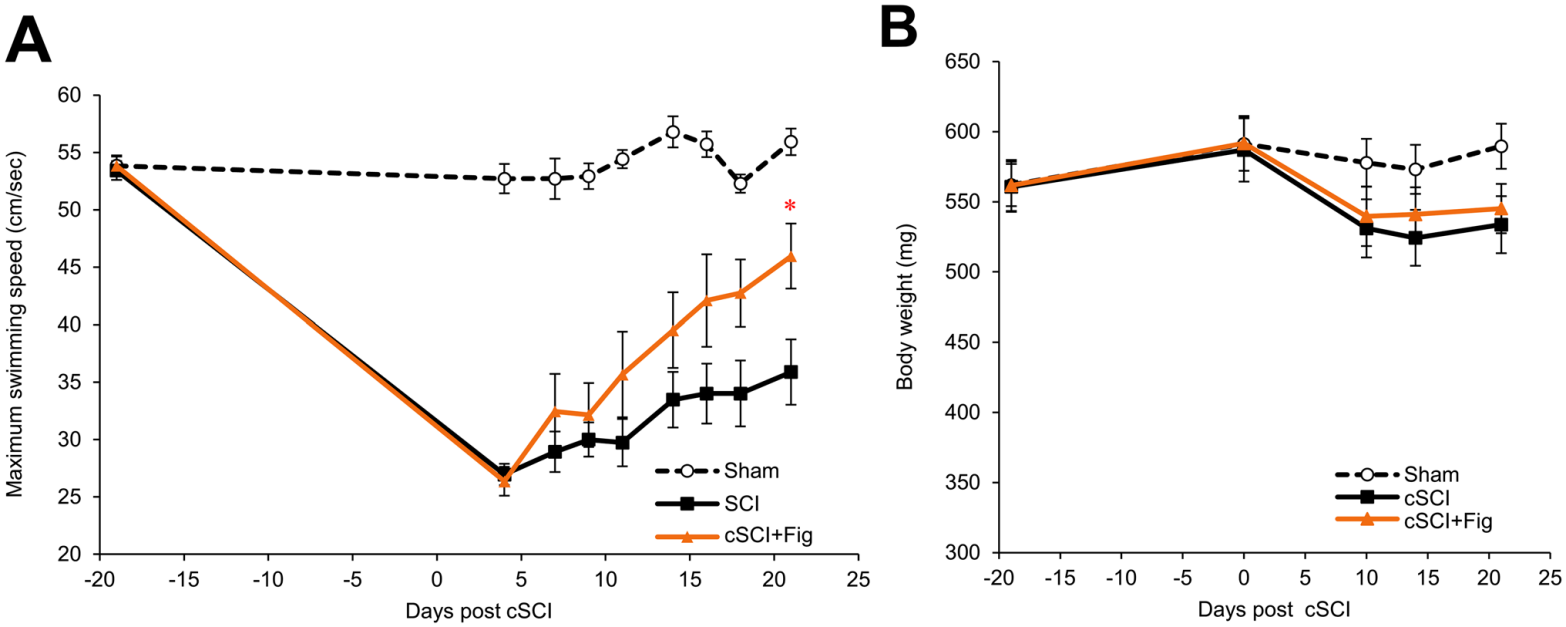
Maximum swimming speed and body weight for the sham, cSCI, and cSCI + fig zebrafish. **a** Time-course changes of maximum swimming speed for the sham, cSCI, and cSCI + fig zebrafish. Maximum swimming speed for the sham, cSCI, and cSCI + fig zebrafish and histological analysis of the neural regenerative effect by fig. **b** Time-course changes of body weight pre- and post-cSCI. Values are expressed as means ± standard errors: *p < 0.05 vs cSCI

In Experiment 4, the cSCI + fig group, which was fed fig-containing bait, was added alongside the sham and cSCI groups. The body weight and maximum swimming speed were measured over time for each group. The maximum swimming speed was markedly decreased in the cSCI and cSCI + fig groups after injury. Subsequently, the swimming speeds of both groups were restored. However, the speed of cSCI + fig group was significantly higher than that of the cSCI group, indicating that the fig extract accelerated functional recovery after cSCI (Fig. 5A). As confirmed in Experiment 2, cSCI treatment prevented the increase in body weight in both groups (Fig. 5B).

### Fig extract administration promotes spinal cord regeneration

**Fig. 6:**
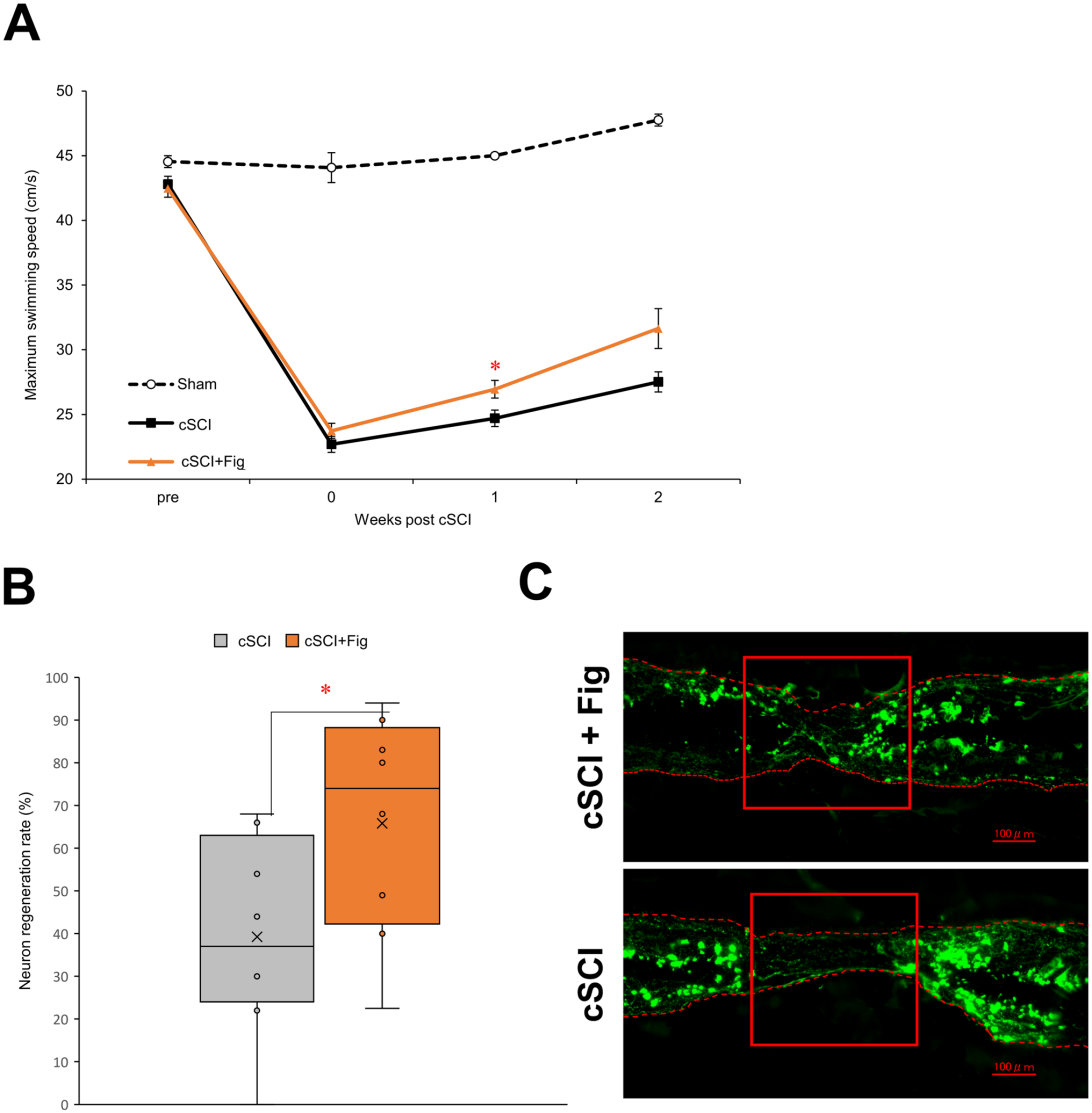
Maximum swimming speed with histological analysis for sham, cSCI, and cSCI + fig treated zebrafish. **a** Time-course changes in maximum swimming speed before and after cSCI. Values are expressed as means ± standard error. **b, c** Neuronal regeneration rate and histological analysis in the cSCI and cSCI + fig zebrafish at 2 weeks post-cSCI. Red squares indicate the cSCI sites. Scale bars in **c** 100 μm. Values are expressed as box plots. Crosses indicate the mean value for each group. The top and bottom edges of the error bars indicate the maximum and minimum values. The top and bottom edges of the box indicate the third and first quartiles. The center line of the box indicates the median value. Each empty circle indicates values for each sample. *p < 0.05 vs cSCI

In Experiment 5, the maximum swimming speed was measured over time for the three groups, followed by dissection at the point when the recovery promotion effect was observed (Fig. 6A). Visualization of *Isl1* expression in Tg zebrafish by fluorescent microscopy showed significant cSCI, whereas the fig-treated group showed promoted regeneration, represented by thicker connections at the damaged site (Fig. 6B, C). The transgenic zebrafish, *Isl-1-GFP*, was approximately 30% smaller than the wildtype and exhibited reduced swimming ability, resulting in a lower maximum swimming speed as indicated in Fig. 6 compared to that in Figs. 3 and 5. Moreover, *Isl-1-GFP* zebrafish had a lower survival rate than the wildtype, prompting us to conduct the experiments twice and integrate the data in Experiment 5.

## Discussion

### Phenotype of cSCI zebrafish model and histological analysis

Spinal cord-injured zebrafish showed markedly reduced maximum swimming speed, which recovered spontaneously. The injured pectoral fin rostral to the lesion is often used and shows short and frequent bursts, resulting in rapid fatigue and low swimming performance (Burris et al. 2021). Although the mean value in the cSCI group was approximately 45% compared with the sham group at day 3 post cSCI, it recovered to approximately 58% at day 10 and approximately 80% at day 18 post cSCI. This was a very rapid reinstatement compared to the spinal cord transection model, which has already been reported (Mokalled et al. 2016).

In addition to zebrafish, teleost fish including goldfish and eels with excellent regenerative abilities have been reported as models of spinal cord transection (Takeda et al. 2024; Doyle and Roberts 2004). However, zebrafish are favored owing to their ease of breeding and genetic manipulation, homology with humans, and the availability of larvae. In assessing spinal cord regeneration, histological analyses are generally performed at the site of injury using cell proliferation and neurogenesis markers (Chang et al. 2021; Wehner et al. 2017), or by evaluating axons and neurons using tracers such as Biocytin (Mokalled et al. 2016). While these evaluation methods are direct and accurate, they are limited by the necessity of dissection, which prevents continuous evaluation. To address these limitations, many studies have been conducted to indirectly assess spinal cord regeneration by measuring behavioral functions. Leveraging the consistent swimming behavior of zebrafish, evaluations based on total distance traveled per unit time or swimmable time for forced swimming have been reported (Becker et al. 2004; van Raamsdonk et al. 1998). Although methods utilizing maximum swimming speed have been documented (Burris and Mokalled 2024; Mokalled et al. 2016), these have primarily involved transection models that sever the spinal cord. In contrast, the model we used in this study involves a damage model that preserves spinal cord connectivity (Hui et al. 2010). This model not only facilitates faster regeneration than the transection model but also better mimics injuries observed in mammals, such as hemisection and contusion injuries. In addition, while functional evaluations based on swimming distance have been previously reported for injury models, assessments of maximum swimming speed have not been explored. The present study is the first to capture the restoration of swimming speed using a crush SCI model. This approach enabled us to capture the decline and recovery of the maximum swimming ability before and after cSCI, while also assessing the effectiveness of food-derived ingredients.

We also revealed that the time course of recovery of the maximum swimming speed by forced swimming and the amount of spontaneous activity by free swimming coincided. According to the regeneration of the spinal cord, the spontaneous locomotor activity also recovered smoothly, and it increased to approximately 40% at day 2 post cSCI compared with the sham group, and to 60% at day 7 post cSCI and 70% at day 17 post cSCI. In contrast, body weight gain was suppressed in the cSCI group after cSCI, which was probably caused by impaired swimming ability. Significant muscle atrophy can cause weight loss and decreased swimming ability, as reported in a zebrafish model of sarcopenia (Chen et al. 2023). A subtle increase in the maximum swimming speed in the sham group might have been induced by repeated swim assays as a type of training. Similar to humans, continuous exercise in zebrafish modulates the expression of growth hormone receptors and myostatin (Palstra et al. 2010) and improves motor function (Heinkele et al. 2021).

We followed the process from injury to spinal cord regeneration by observing *Isl1*, a motor neuron marker, in transgenic zebrafish. We confirmed that the connecting part gradually became thicker after the postoperative injury, and completely recovered to the same level as that in the sham group on days 22 and 35 post-cSCI. Furthermore, the myelin marker *Mbp* was regenerated. Because the connection at the injury site was torn at day 0 post-cSCI, it completely recovered by day 35 post-cSCI. In the transection model, debris is removed 3 days postoperatively and tissue cross-linking of the injured area occurs 15 days postoperatively (Cigliola et al. 2020). Adequate histological reconnection requires more than 6 weeks after the operation (Mokalled et al. 2016). These data suggest that the present cSCI model showed more rapid neural regeneration than the transection model.

Taken together, we confirmed that histological spinal cord regeneration and recovery of swimming function occur simultaneously in the cSCI model, as reported in the transection model (Huang et al. 2021; Mokalled et al. 2016); thus, maximal swimming speed is an indirect indicator of spinal cord regeneration in adult zebrafish.

### Effects of fig administration on promoting spinal cord regeneration

The recovery of maximum swimming speed after cSCI in zebrafish was promoted by the administration of a fig-containing diet. There were no differences in food intake or body weight owing to fig consumption, and we consider that the facilitation of swimming speed recovery was mainly derived from the effects of fig components. The maximum swimming speed and histological neural regeneration were evaluated. Histological assessments of the thickness of neuron markers at the injured site were performed when fig efficacy was confirmed at the maximum swimming speed, resulting in significantly promoted spinal cord regeneration. Overall, the administration of fig extract to zebrafish showed a remarkable neural regenerative effect, similar to that reported in a neuropathy mouse model (Sugita et al. 2024). This may be because the peripheral nerves of rodents and the central nerves (spinal cord) of zebrafish share partial regenerative mechanisms. Therefore, fig extract might contribute not only to peripheral nerve damage but also to the regeneration of the CNS in mammals, making it a potential target for drug discovery.

Future studies should examine the mechanism of fig-induced spinal cord regeneration by using this evaluation method. Our previous study showed an increased expression of the macrophage marker *F4/80* in the peripheral nerves of neuropathic mice (Sugita et al. 2024). Macrophages contribute substantially to spinal cord regeneration in zebrafish. After cSCI, macrophages play a role in regulating glial scar formation by promoting the removal of myelin debris and release of chondroitin sulfate proteoglycans and other factors that inhibit excessive scar formation (Zeng 2023). To clarify the discrepancy between these results, it is important to comprehensively examine the macrophage population using flow cytometry or M1/M2 markers. In addition, whether macrophages intervene in the neural regeneration-promoting effects of fig requires more detailed studies using transgenic macrophage-deficient zebrafish.

### Significance of present evaluation method system as a rodent alternative and industrial application

In the present study, we investigated zebrafish spinal cord, which shares mechanisms with rodent peripheral neurons, as an alternative to rodent models that assess nerve regeneration. The effectiveness of fig extract, which has a strong nerve regeneration effect on the peripheral nerves in mice, has also been confirmed in the spinal cord of zebrafish. Three important characteristics of the present evaluation system are as follows. First, adult zebrafish, but not larvae, were used to perform swim capacity assays and histological analysis at an in vivo level. Secondly, the cSCI model, but not the transection model, was used to increase the postoperative survival rate and speed of recovery. Third, the custom-made water 320 L tank is based on a seawater tank for hydrodynamic experiments, which can assess up to 16 fish with two courses simultaneously, generate a uniform water flow, and enable a stable and high-throughput evaluation system.

Compared to the mice, the zebrafish evaluation system is superior in industrial application. In terms of time and efforts, much less time is required for a single test. Mouse assays need approximately 4–5 weeks until the effect of the material is confirmed by motor neuron conduction velocity or histological analysis. In contrast, zebrafish need only 2–3 weeks, and the present evaluation method measures only swim capacity. Additionally, the mice required isolation in separate cages during pain assays for protection against attacks from each other, whereas 16 zebrafish were housed in a single small cage. Regarding cost, fewer materials are required for zebrafish assays. In the fig extract experiment, a mouse needs 40 mg/day of the fig extract, whereas a zebrafish only needs 0.3 mg/day. This difference offers a significant advantage when conducting larger-scale screening.

However, this study has several limitations. First, the compatibility of this evaluation system with mammals. This system evaluates the regeneration of the zebrafish spinal cord. Although it shares some mechanisms, its compatibility with peripheral nerve regeneration in mice and humans is debatable. Second, the stepwise increase in swimming speed, which was set at 1 Hz/min for throughput, but a smaller value may provide a more reliable value. Third, the method of administering the material. We administered by mixed feeding, and since the material may have leached into the water, the oral method may be more suitable if the dosage is strictly regulated. Finally, the variations in swimming speed recovery and the timing of the onset of the material’s effects differed from assay to assay. This variability was primarily attributed to differences in the extent of damage caused by cSCI performed with forceps. Within the same experiment, the degree of damage was adjusted by establishing exclusion conditions; however, for comparisons between assays, the relative values compared to the cSCI group remain important for interpretation.

In summary, this novel evaluation system enabled us to obtain similar results to those of mice with high reproducibility. This system will also enable quicker and more cost-effective evaluation of materials and will contribute to reducing the number of experiments using rodents. Future investigations may identify the active ingredients of fig extracts using this evaluation system.

## Conclusions

In this study, we developed a novel nerve regeneration assessment system using adult zebrafish with cSCI as an alternative to rodents. The effectiveness of fig extract, a natural compound that promotes neural regeneration, was confirmed. This evaluation system is superior in cost and throughput compared to tests with rodents, and it could evaluate physiological activity which is not feasible by in vitro screening. Hence, the present system is expected to be applied to drug discovery and healthcare product development.

## Data Availability

No datasets were generated or analysed during the current study.

## Declarations

### Employment

HM, SS, YH, TH, SM, NO, and YM are employed by Kao Corporation.
